# Quantitative Analysis of Composite Polymeric Membrane Structure Using Contour Morphological Heterogeneity Functions

**DOI:** 10.3390/polym18151831

**Published:** 2026-07-27

**Authors:** Georgy Rytikov, Fedor Doronin, Yuriy Rudyak, Andrey Evdokimov, Alexander Tarasov, Victor Nazarov

**Affiliations:** 1 Faculty of Printing Industry, Moscow Polytechnic University, 107023 Moscow, Russia; 2 Technofilter Research–and-Manufacturing Enterprise, 600031 Vladimir, Russia

**Keywords:** separating, filtering, membrane architecture, structural heterogeneity, spatial distribution, surface, bulk and transition layers, scanning electron microscopy, image quantitative analysis, computer vision, contour morphological heterogeneity functions

## Abstract

We have developed a quantitative method for characterizing the actual multilayer polymer material-made membranes’ architecture with a computer analysis of their scanning electron microscopy (SEM) images. The proposed approach consists of contour morphological heterogeneity function (CMHF) calculations. The approbation was carried out on polysulfone, polytetrafluoroethylene and polyimide-made membranes. The corresponding CMHF series were computed for different depths. It was possible to identify the structural zones’ location for all the considered membranes. The developed technique provides the possibility of standardizing the SEM images’ analysis and determining the thicknesses of the surface, transition and volumetric layers of filtering and separating equipment elements. The suggested algorithm can automate the membrane’s structure and architecture metrological analysis with computer vision techniques.

## 1. Introduction

The goals of modifying the elements of filtering and separating equipment are either to give them improved properties or to significantly reduce the cost of their production (while maintaining the level of quality indicators unchanged) [1,2,3,4]. According to the basic paradigm of materials science, the properties of high-tech (usually composite) membranes and filters are determined by their morphology (elemental composition and chemical and physical structure) [5,6,7].

It has a decisive effect on the permeability, the efficiency of mixture components’ separation, the strength and the duration of the items’ operation [8,9,10]. For example, according to [11,12], the structure and the properties of the surface are key complex factors determining the efficiency of membrane gas separation (natural gas treatment, extraction of carbon dioxide from industrial exhaust, purification of helium, etc.).

The porosity (shape, size, and type of pore distribution) directly determines the balance between permeability and selectivity of filtering and separating equipment [13,14,15]. For example, [13,16,17] mention that the asymmetric membrane architecture with a thin dense separation layer and a porous durable base reduces the product filtration resistance and increases the device permeability. In this case, the permeability depends on the selective layer thickness (the thinner the layer—the higher the permeability). This is due to the overall resistance to diffusion through the membrane decreasing.

The surface morphological features affect the effectiveness of the material interaction with the separated components’ medium. They can change the miscibility of the surface and/or the hydrodynamic resistance to liquid or gas flowing, hinder or facilitate the deposition of particles, etc. [18,19]. The movement of mixed flow near the membrane surface depends on the distribution of textural inhomogeneities that determines the separation frontal uniformity. The surface heterogeneity increasing affects the filters’ sorption and the permeability [20,21,22]. The modification (for example, by applying thin layers of polysiloxane, fluoropolymers, other materials or by the gas-phase treatment [23,24,25]) makes it possible to change the structure and, as a result, the surface layer’s physico-chemical properties (hydrophilicity, adsorption capacity, etc.) for the membranes [26,27].

It is usually necessary to achieve high accuracy in the materials’ distribution across the layers when producing the membranes with layered architecture (especially in the case of composite and/or multilayer item manufacturing [28,29,30]). The structural components have to ensure the mechanical stability of the filtration system; the functional ones have a high level of selectivity. The surface defects or inhomogeneities of the polymer membranes can initiate local concentrations of mechanical stresses, which reduces the overall reliability of the industrial filters [31,32].

Scanning electron microscopy (SEM) is a widely used method for visualizing the structure of various types of materials [33,34,35,36,37,38,39]. The main technical SEM advantage is the high spatial resolution, which makes it possible to observe microcracks, pores, inclusions, and other inhomogeneities that cannot be studied by optical microscopy [33,34].

SEM is used both in laboratory research (for example, in microporous and nanostructured polymer membranes analysis, when studying nanofiltration and ultrafiltration material systems, and in the case of ion exchange membrane material investigation) and in industrial samples’ quality verification [35,36].

Electron microscopy techniques are also used to visualize the surface damage, to identify the stress concentration zones and the type of fracture (brittle, viscous, layered), to detect the structural inhomogeneity affecting the product’s strength (for example, during the membranes and filters mechanical tests) [35,36,37].

The experimental samples’ cross-sections’ SEM images are often used to demonstrate the achievement of separating products’ certain characteristics [38,39]. In some cases, the surface layer thickness can be estimated by a small number of measurements (the set cannot be considered as “representative”) and without taking into account the features of the observed spatial texture heterogeneity distribution. Apparently, this is due to the lack of a technique by which it would be possible to determine where the surface “ends”, whether there is a “transition layer” and where the volume of the membrane “begins”.

We propose to adapt our previous approach to the packaging (insulating) polymer films’ and needle-punched (filtering) nonwoven fabrics’ SEM images’ quantitative description for determining the architecture parameters of various polymer material-made multilayer composite membranes. Its key feature is the possibility of correlating the contour heterogeneity functions at special points with the position of the composite filters and membrane layers’ centers of mass levels.

## 2. Materials and Methods

We have previously developed and tested a tomographic concept for the quantitative characterization of high- and low-porous polymer materials’ surface structure heterogeneity [40,41]. The essence is to compare a sample surface fragment with a series of images binarized by a threshold value of pixel brightness, calculating the total boundary length of the observed flat structures. The latter characterize the sample surface microrelief “bulge” and “concavity” distribution.

### 2.1. Morphological Heterogeneity Functions

The contour morphological heterogeneity function λ(n) is a SEM image fragment perimeter-normalized dependence of the pixels’ brightness constant level line length on the corresponding threshold value n:

(1) $$\lambda \left(n\right)=\frac{1}{L_{0}(B(x,y))}\cdot \sum_{k=1}^{K(n)}l_{k}(n)$$

**Figure 1:**
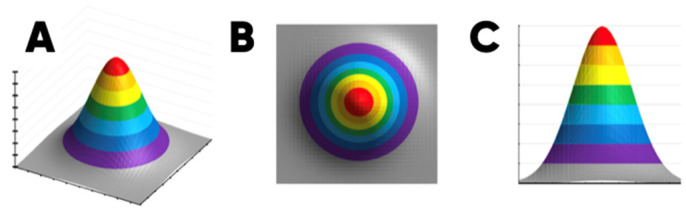
The 8-level Gaussian model of an ideal protrusion—(**A**), its frontal—(**B**) and profile—(**C**) projections.

Here B(x,y) is a digital representation of an analyzed SEM image fragment with $L_{0}$-perimeter; K(n)—the number of closed shapes ${\left\{b_{k}(x,y)\right\}}_{K\left(n\right)}$ formed when B(x,y)n-threshold binarizing; {${\left\{l_{k}(n)\right\}}_{K\left(n\right)}$}—the set of $b_{k}(x,y)$-boundary lengths. A “digital image” (in the information-logical sense) is a tabulated function B(x,y) that reflects the dependence of pixel brightness B on its location coordinates (x,y). And it can be characterized by a perimeter $L_{0}=2\cdot \left(x_{max}+y_{max}\right)$ where $x_{max},\;y_{max}$—the coordinates with maximum values. Let us consider the 8-level Gaussian geometric model of an ideal protrusion above a perfectly smooth surface, its frontal and profile projections (Figure 1).

The same color indicates the area where the pixels’ brightness of the corresponding (SEM) image would be the same. If we choose the binarization threshold n to be 1, 2, 3, 4, 5, 6, and 7, then the corresponding closed shapes $b_{k}(x,y)$ and their boundaries $l_{k}(n)$ will take the forms shown in Table 1. The $\lambda \left(n\right)$-means are calculated by Equation (1).

**Figure 2:**
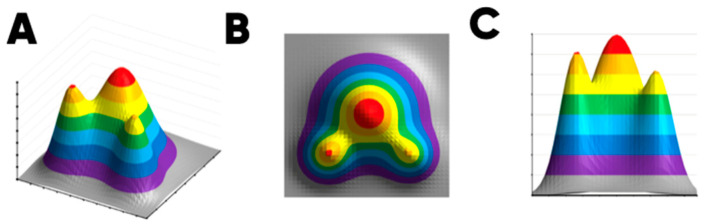
Three-Gaussian surface local inhomogeneity model: 3D—(**A**), frontal—(**B**) and profile—(**C**) projections.

If there are several Gaussian protrusions with different characteristic sizes above a perfectly flat surface, then the binarization will result in some (K(n)) regions bounded by closed contours (Figure 2).

The corresponding closed shapes and boundaries are presented in Table 2.

In general, a 256-level digital real surface model (SEM image) is similarly matched with a set of binarized primitives (shapes and boundaries).

**Figure 3:**
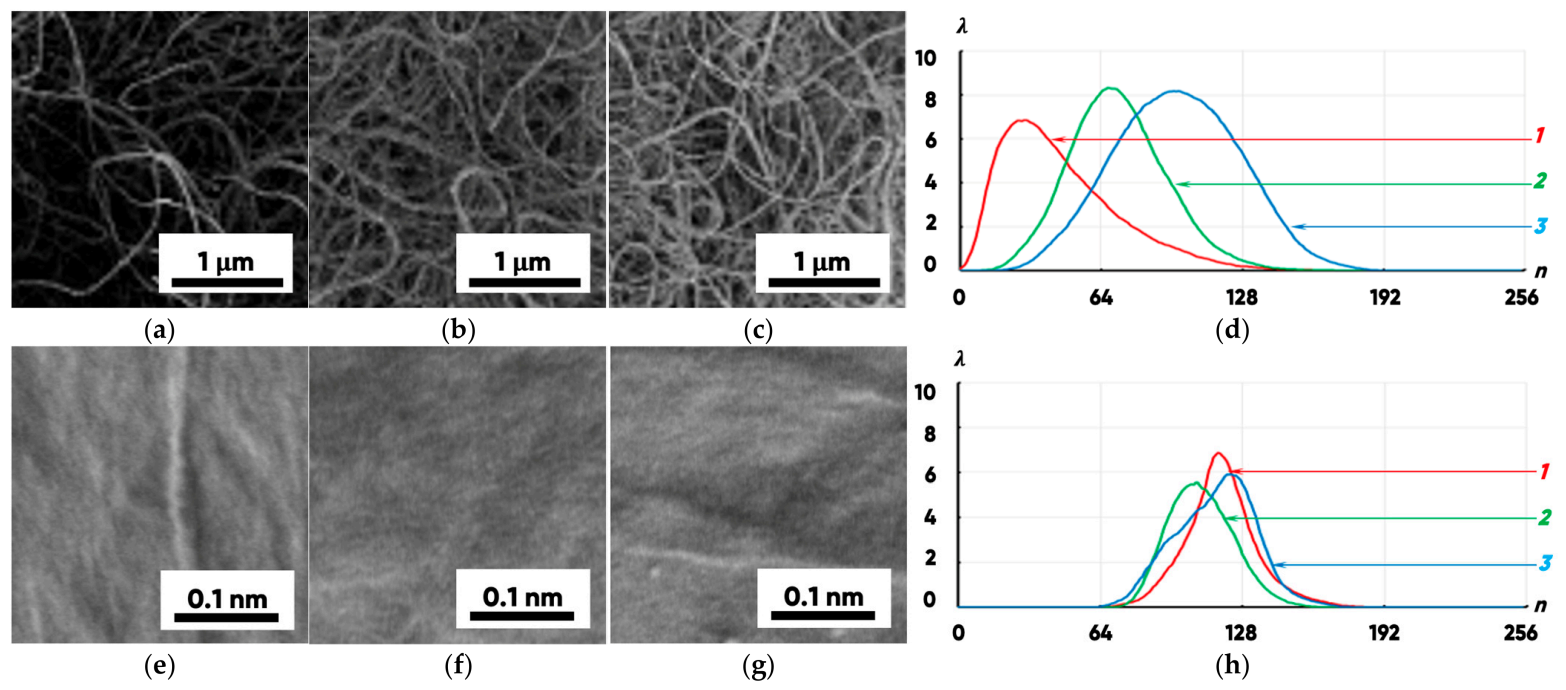
The frontal SEM image fragments of fibrous nonwoven fabrics—(**a**–**c**) and polymer films [40,41]—(**e**–**g**) with corresponding contour morphological heterogeneity functions (CMHFs)—(**d**,**h**). The numbers 1, 2, 3 indicate the CMHF curves respectively characterizing (**a**–**c**) and (**e**–**g**).

Figure 3 shows the frontal SEM image fragments of fibrous nonwoven fabrics [40] and packaging polymer films [41] with corresponding contour morphological heterogeneity functions (CMHFs) $\lambda \left(n\right)$.

The characteristic curves (obtained using the original software (RU2024685170) differ significantly from each other for samples of fibrous nonwoven fabrics made at temperatures of 20, 130 and 190 °C (for example, by the position of the maximum or by the width at half-height level). And the CMHF for different SEM image fragments of low-density polyethylene-made packaging film differs slightly. Thus, the contour morphological heterogeneity function λ(n) is sensitive to the experimental samples’ structural inhomogeneities. But it is not probabilistic, which in some cases makes it difficult to interpret the results obtained during the analysis.

We introduce the integral function of contour morphological heterogeneity Λ(n) as a result of maximum-normalized cumulative addition of λ(n)-values set:

(2) $$\Lambda \left(n\right)=\frac{1}{max(\Lambda (n))}\cdot \sum_{n=0}^{255}\lambda_{n}$$

Such a function will be equivalent to the empirical probability of the constant level line (not necessarily simply connected) with a total length L not exceeding the $\lambda \left(n\right)\cdot L_{0}$-value (here $L_{0}$ is the analyzed image fragment perimeter). The $\Lambda \left(n\right)$ is non-decreasing $\left(\forall n_{1}<n_{2}:\;\Lambda \left(n_{1}\right)\leq \Lambda \left(n_{2}\right)\right)$ and constrained $\left(\;\Lambda \left(0\right)=0;\Lambda \left(n_{max}\right)=1\right)$.

**Figure 4:**
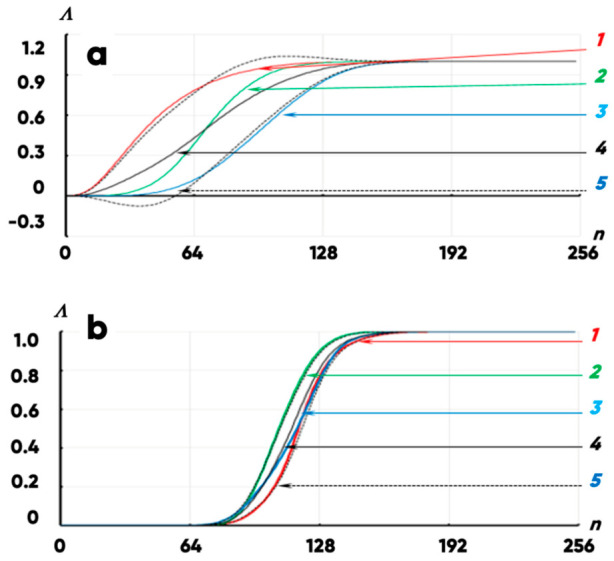
The integral contour morphological heterogeneity functions of PET-fiber nonwoven fabrics made at temperatures of 20 (**a**:1), 130 (**a**:2) and 190 °C (**a**:3) and for three randomly selected surface fragments of the same LDPE film (**b**:1,2,3). Continuous black lines indicate the results of averaging (4); dashed lines indicate confidence intervals at the ~0.7 level (5).

Figure 4 shows the integral contour morphological heterogeneity functions (ICMHFs) calculated (2) for the PET-fibrous nonwovens (Figure 3a–c) and LDPE films (Figure 3e–g).

It is clearly seen (Figure 4a) that the integral functions of contour morphological heterogeneity for the PET-fiber nonwovens manufactured at different calendering temperatures differ significantly from each other (the average value of the standard deviation is ~0.11). At the same time, different fragments of the same LDPE packaging material are characterized by curves set with a standard deviation (~0.02) (Figure 4b). This is 5.5 times less than in the case of the nonwoven fabric samples with significantly different technological histories.

It should be noted that the integral morphological heterogeneity function averaged over an ensemble of membrane or filter surface image fragments can be considered as a vector characterizing the surface structure of the material from which the item is made. The corresponding representation will make it possible to use the introduced structural characteristic for automated image analysis with computer vision and/or image recognition techniques in the future.

### 2.2. Various Conditions Testing

Several different-scale images of filtering equipment composite elements were selected from literary sources to test the proposed technique on arbitrary samples. In particular, the proposed approach was applied to the quantitative description and analysis of the cross-sectional images of the polymer membranes made on the basis of polysulfone, polytetrafluoroethylene and polyimide [13,42,43].

The digital twins of the membranes’ cross-sections were divided into fragments corresponding to different analysis depths. The contour heterogeneity functions were calculated for each computational window obtained. And they distinguish to a greater or lesser extent (depending on the conditions of the numerical experiment) in shape and values of key characteristics (abscissae and ordinates of extremes and inflection points, boundaries of the range of variation, etc.).

The membranes considered differed significantly from each other in geometric dimensions, architecture, and physical structure. However, in all cases, the proposed technique allowed us to form a series of characteristic functions with which the morphology of the product can be described quantitatively (using multidimensional vectors $\lambda \left(n\right)$ or $\Lambda \left(n\right)$).

#### 2.2.1. Membrane Structure Scale: 100–1000 Microns

**Figure 5:**
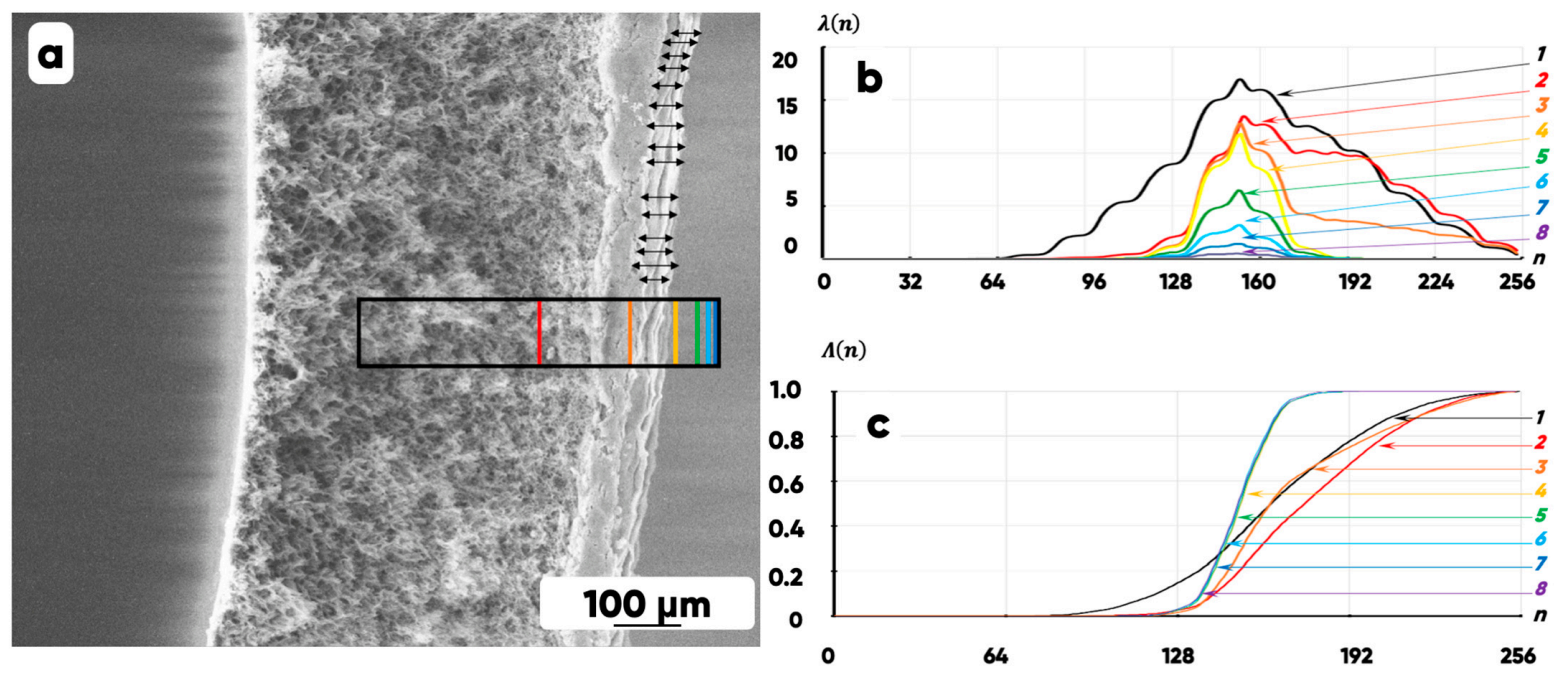
The results of quantitative characterization of polysulfone-based hollow fiber membrane [42] physical structure: SEM image—(**a**) (the “volume” layer is on the left, the surface one is on the right); contour morphological heterogeneity functions λ(n)—(**b**) and Λ(n)—(**c**). The curves 1, 2, 3, 4, 5, 6, 7 and 8 are plotted for the SEM image fragments indicated by colored rectangles in (**a**). The boundaries of the computational window with 512 microns’ depth analysis are black; the ones for 256, 128, 64, 32, 16, 8, and 4 are red, orange, yellow, cyan, blue and violet respectively. The arrows indicate the positions where the layer thickness was measured manually.

The item physical structure in the scale range of 100 to 1000 microns was characterized using the example of a polysulfone-based hollow fiber membrane [42] (Figure 5). The results of polysulfone-based hollow fiber membrane surface layer direct measurements are presented in Table 3.

The well-observed oscillations (12 local maxima) in Figure 5b indicate that at low SEM image magnifications (~100 times) several plans (foreground, background, and 10 intermediate ones) should be distinguished in order to correctly quantify the morphology of the sample. Moreover, the information noise (the deviations of pixels’ brightness which do not carry the information about the hollow fiber cross–section structure; curves 4–8) is so large that five plans (according to the number of local maxima in the range of variation ($n\in \left[128;192\right]$) for the lowest curve 8) should be reserved for its detailed characterization. The remaining 7 (12 local maxima in total, 5—for the noise description) plans can be used to identify the boundaries of the composite membrane layers. The calculation and visualization (Figure 5c) of the integral contour morphological heterogeneity functions (ICMHFs) makes it possible to clearly distinguish four types of curves characterizing the presence on an image fragment of the: background noise (curves 8–4), surface (curve 3), transitional (curve 2) and volumetric (curve 1) layers. Thus, by varying the width of the computing window (correlated with the depth of immersion in the membrane thickness) and observing the change in shape and position of the key ICMHF characteristics (range of variation, average tangent of the tangent angle, etc.) it is possible to accurately determine the “passage” of the digital probe boundary between the layers of the product and, as a result, to standardize the procedure for identifying the actual multilayer membranes’ architecture.

#### 2.2.2. Membrane Structure Scale: 10–100 Microns

**Figure 6:**
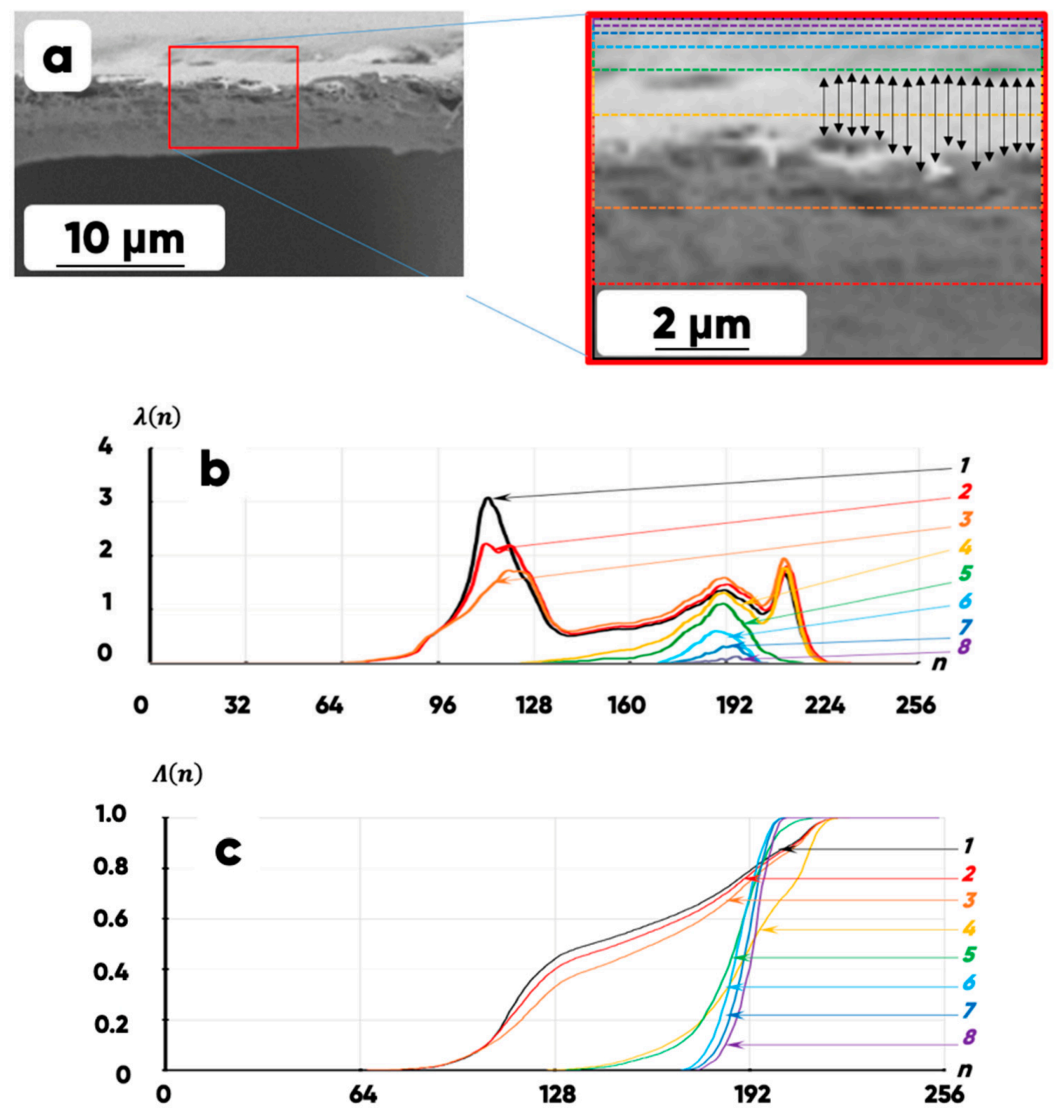
The results of physical structure quantitative characterization for the composite membrane, the functional layer of which was made of sulfonated polytetrafluoroethylene [43]: SEM image—(**a**) (the volume layer is “bottom”; the surface one is “top”); contour morphological heterogeneity functions λ(n)—(**b**) and Λ(n)—(**c**). The curves 1, 2, 3, 4, 5, 6, 7 and 8 are plotted for SEM image fragments indicated by colored rectangles in (**a**). The boundaries of the computational window with 8 microns’ depth analysis are black; the ones for 6, 4, 2, 1, 0.5, 0.25 and 0.125 are red, orange, yellow, green, cyan, blue and violet respectively. The arrows indicate the positions where the layer thickness was measured manually.

The membrane physical structure’s quantitative characterization in the scale range from 10 to 100 microns was performed using the example of a sample consisting of a sulfonated polytetrafluoroethylene functional layer and a polyvinylene fluoride copolymer substrate [43] (Figure 6). The results of direct measurements of the transition layer between the sulfonated polytetrafluoroethylene and polyvinylene fluoride copolymer substrate are presented in Table 4.

The set of oscillations (as in Figure 5b) is no longer observed in this case (Figure 6b). As a result, the three “main” planes (well correlated with intuitive concepts of the surface, transition and bulk layers of the membrane) can already be identified unambiguously by the number of the most pronounced local extremes of the CMHF $\lambda \left(n\right)$ (curves 1–3) itself. At the same time, it is obviously more convenient to use the integral contour morphological heterogeneity function (ICMHF) for both visual (Figure 6c) and quantitative similarity analysis of samples’ structure with different computational window depths. The ICMHF curves 1–3, 4, 5 and 6–8 clearly form three clusters. The shape of curve 5 shows that in $n\in \left[128;192\right]$-region it is similar to curve 4 but in the $n\in \left[192;224\right]$-region—to curves 6–8. It is due to the spatial heterogeneity of the analyzed surface micro-roughness. Thus, the “upper boundary” of the membrane should be considered at level 5 (“green”). The boundary of the “transition layer” is ~1 micron away from it (“yellow”). And the structure of the polymer matrix of the product substrate can be observed starting from a depth of ~4 microns (“orange”).

#### 2.2.3. Membrane Structure Scale: 1–10 Microns

**Figure 7:**
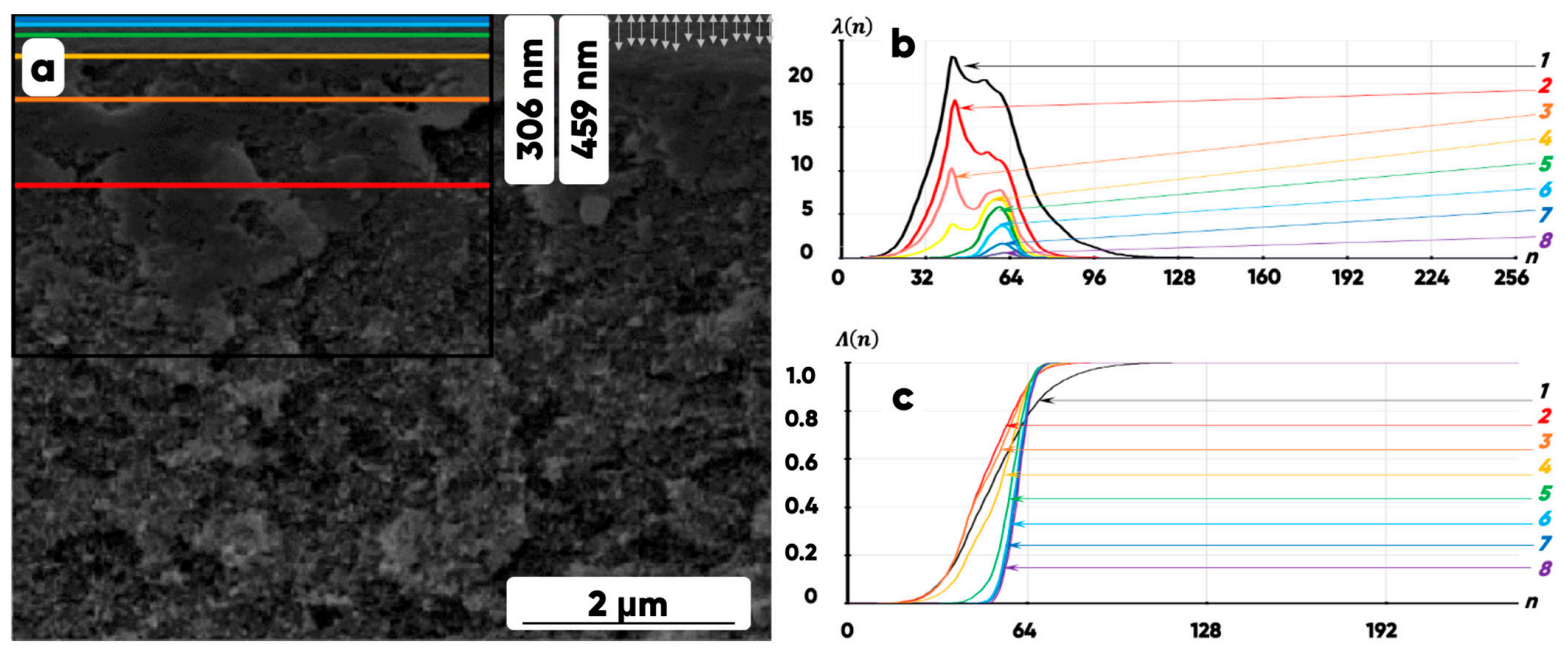
The results of polyimide copolymer-based hollow fiber composite membrane [13] physical structure quantitative characterization: SEM image—(**a**); contour morphological heterogeneity functions λ(n)—(**b**) and Λ(n)—(**c**). The curves 1, 2, 3, 4, 5, 6, 7 and 8 are plotted for SEM image fragments indicated by the colored rectangles in (**a**). The boundaries of the computational window with 4.0 microns’ depth analysis are black; the ones for 2.0, 1.0, 0.5, 0.25, 0.125, 0.0625 and 0.03125 are red, orange, yellow, cyan, blue and violet respectively. The authors’ [13] results of layer thickness measurement are in white rectangles.

The membrane physical structure characterization in the scale range of 1 to 10 microns was made for the polyimide-based hollow fiber [13] (Figure 7). The results of polyimide-based hollow fiber membrane surface layer direct measurements are presented in Table 5.

Figure 7b shows that there is only one characterizing membrane surface single peak (curves 8–5) when the analyzed layer thickness varies from 0.03125 to 0.25 microns. A second peak appears (curves 4–1) when the depth grows from 0.5 to 4.0 microns. Obviously, the heights of these peaks can be almost the same with a certain value of the analysis depth. It can be used to automatically identify the location of the boundary between the “surface” and the “volume” of the membrane. The first peak (at *n* = 60) (which characterized the surface of the sample exclusively) practically ceases to be observed against the general background as the analysis depth becomes 4 or 2 microns (curves 1 and 2). But another “new” weak peak appears (at *n* = 56). That is probably indicating the possibility of a transition layer existing in the membrane structure (between the “surface” and “volume” ones).

As expected, it follows from Figure 7c that the information-significant threshold pixel brightness variation interval increases (and the average tangent angle decreases) the more the sample thickness falls into the digital analysis window. The surface boundary of the membrane is located at level 5 (“green”); the boundary of the transition layer corresponds to level 4 (“yellow”). The observed significant spatial heterogeneity of the polymer matrix structure leads to a statistically significant difference between curve 1 (“black”) and curves 2 and 3 (“orange” and “red” that form a cluster). Thus, the thicknesses of the surface and the transition layers can be estimated at 0.25 and 0.5 microns, respectively. And statistically significant differences in the morphology of the polymer matrix volume will be observed at an analysis depth exceeding 2 microns.

## 3. Results and Discussion

We have manufactured the prototypes of polyethyleneterephthalate-based composite filters and membranes. The filters were made from PET fibers using a hot calendering process at a temperature of 160 °C and a rolling speed of 6 m/min. The possibility of filter-architecture regulating with the mechanical stretching was demonstrated. The membranes were extruded from PET granules and subjected to gas-phase treatment with fluorine- and oxygen-containing gas mixtures (to control the permeability of the products to n-heptane).

### 3.1. The Filter Prototype Analysis

**Figure 8:**
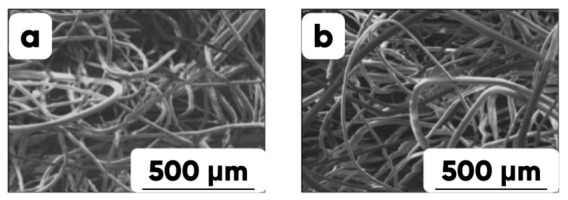
The SEM images of fragments of the PET-based composite filters [40] cross-section: initial—(**a**) and 10-elongated—(**b**).

We have investigated how the tensile deformation affects the PET-filter architecture by analyzing changes in contour morphological heterogeneity functions. Figure 8 shows the optical images’ fragments of the PET-based composite filters cross-section: “initial” (Figure 8a)—made from PET fibers by hot calendering in an air atmosphere at a temperature of 160 °C and a rolling speed of 6 m/min, and “elongated” (Figure 8b)—subjected to the longitudinal stretching 10-deformation.

The felt filter morphology changes much more significantly than the bulk one under the thermomechanical processing. A denser (reinforced) layer with lower porosity is formed as a result of induced near-surface fiber fusion. While the volume component retains a relatively high permeability for a wide range of liquids of various chemical compositions. However, the concept of “surface” and “layer” is not well defined for highly heterogeneous fibrous materials. As a result, when analyzing (using optical, electronic, atomic force, and other microscopy techniques) the structure of the experimental samples for the subsequent new products’ architecture development, a high degree of ambiguity arises in the interpretation of the obtained images’ information (in context of determining the location of boundaries between “surface”, “transition” and “bulk” structural components). At the same time, the main properties (strength, porosity, permeability, etc.) of the considered composite items depend on the “thicknesses” of the corresponding “layers.” Thus, we have developed a method for establishing physically poorly identifiable interlayer boundaries based on the ensemble analysis of contour morphological heterogeneity functions.

**Figure 9:**
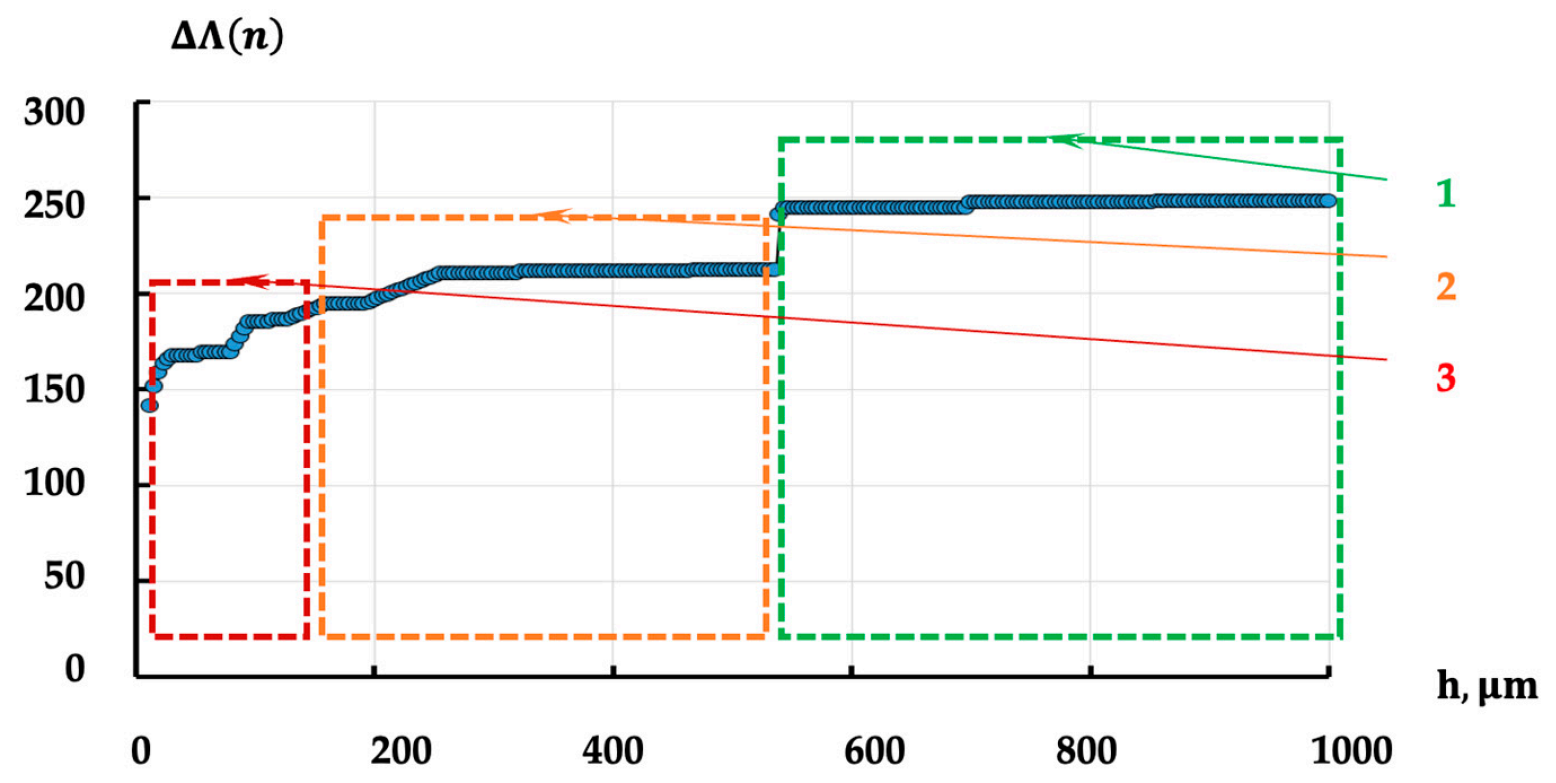
The dependency of the ICMHF-variation width $\Delta \Lambda \left(n\right)$ on the depth h,μm of sample analysis for the initial filter prototype (made by PET fibers calendered at 160 °C temperature and 6 m/min rolling). The “surface” is on the left.

**Figure 10:**
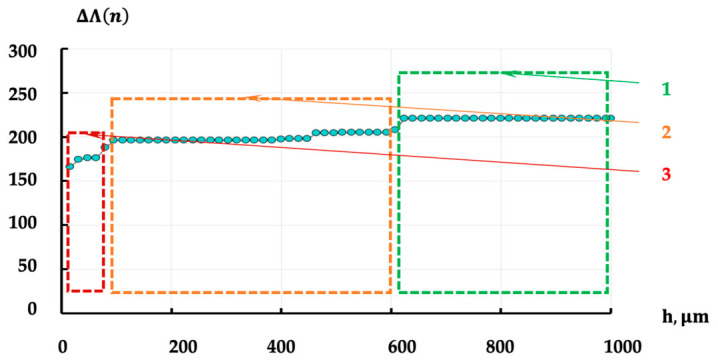
The dependency of the ICMHF-variation width $\Delta \Lambda \left(n\right)$ on the depth h,μm of sample analysis for the 10-elongated PET-fiber calendered filter. The “surface” is on the left.

We have calculated the ICMHF-variation ranges’ widths for the samples’ cross-section images’ fragments that characterize different depths of the filter prototype profiles’ analysis. The obtained width vs. depth dependencies for the initial and elongated samples are presented in Figure 9 and Figure 10.

The “plateaus” (“steps”) corresponding to the “bulk” (1), “transition” (2), and “surface” (3) structural components of the filter prototype are visible in Figure 9. And we have additionally implemented the standard k-means technique (KMeans from sklearn.cluster (Python) scikit-learn 1.9.0) to standardize and automate the boundary identification procedure.

It can be seen that the surface (3) layer thickness decreased from 144 to 76 (±8) μm while the transition (2) one increased from 394 to 538 (±8) μm as a result of elongation. That is in good agreement with the deformable solids continuity.

### 3.2. The Membrane Prototype Analysis

**Figure 11:**
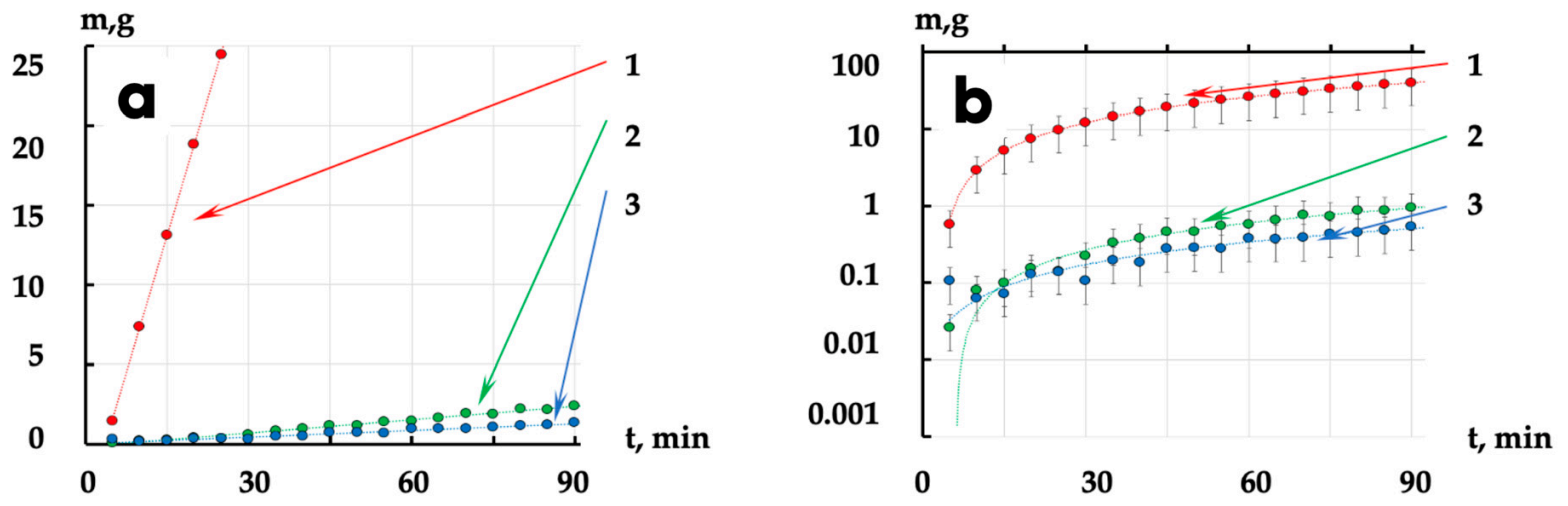
The dependences (in linear—(**a**) and in logarithmic—(**b**) scales) of n-heptane permeate mass m,g on the n-heptane exposure time t, min for the initial—(1), fluorinated—(2), and oxyfluorinated—(3) samples of manufactured low-porous PET-membrane prototypes.

We have investigated the effect of the fluorine- and oxygen-containing gas mixtures’ treatment on the morphology and permeability of the manufactured low-porous PET-membrane prototypes. Figure 11 shows the dependence of the n-heptane permeability on the “in solvent” exposure time for the samples: initial (1) and treated (2, 3) by gas mixtures $He/O_{2}/F_{2}=85.5/0.5/14$ and $He/O_{2}/F_{2}=85.5/6.5/8\mathrm{vol}.\%$ to achieve the average gravimetric modification degree (GMD) of $≅80\;mg/m^{2}$ (i.e., within ~30 and ~50 min, respectively). GMD is the modified object integral mass increase divided by its surface area.

In the context of PET membranes’ property-directed regulation, oxyfluorination increases hydrophilicity and can enhance the adsorption of separated mixtures’ polar components (i.e., the selectivity changes).

The SEM images of the manufactured PET membranes (original, fluorinated, and oxyfluorinated) prototypes’ cross-sections were obtained to determine the thicknesses of the formed integrated fluorine- and oxygen-containing coatings. And a set of corresponding contour morphological heterogeneity functions for the corresponding representative 2 μm × 2 μm fragments was calculated. All averaging was performed over ensembles of 5 different representative fragments of the analyzed SEM images.

**Figure 12:**
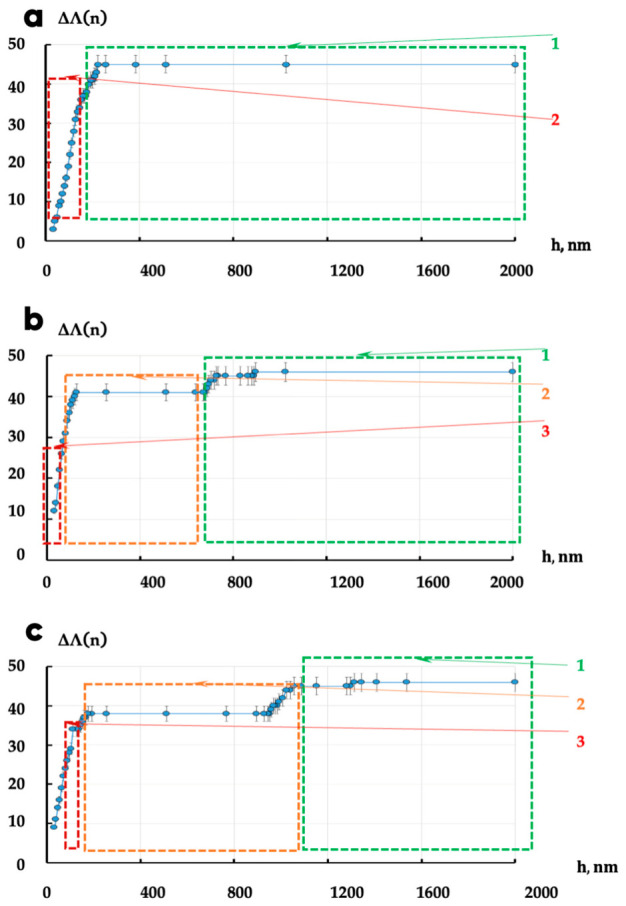
The dependencies of the average ICMHF-variation width $\Delta \Lambda \left(n\right)$ on the depth h,nm of digital analysis for the initial—(**a**), fluorinated—(**b**) and oxyfluorinated—(**c**) low-porous PET-membrane prototypes.

The calculated dependencies of the average ICMHF-variation width $\Delta \Lambda \left(n\right)$ on the depth h,nm of digital analysis for the initial (Figure 12a), fluorinated (Figure 12b), and oxyfluorinated (Figure 12c) low-porous PET membrane prototypes are presented in Figure 12.

Only two clusters with an interlayer boundary at 92(±6) nm were identified when studying the architecture of the initial (unmodified) samples. It can be used to quantify the thickness of the surface layer of PET membranes. And it was found that both (fluorinated and oxyfluorinated) cases resulted in “transition” (integrated functional fluorine- and oxygen-containing) layers with widths of 606 and 896(±8) nm, respectively. As follows from Figure 11 and Figure 12, a greater thickness of the “transition” layer (Figure 12c) corresponds to a lower n-heptane permeability (curve 3 in Figure 11b).

**Figure 13:**
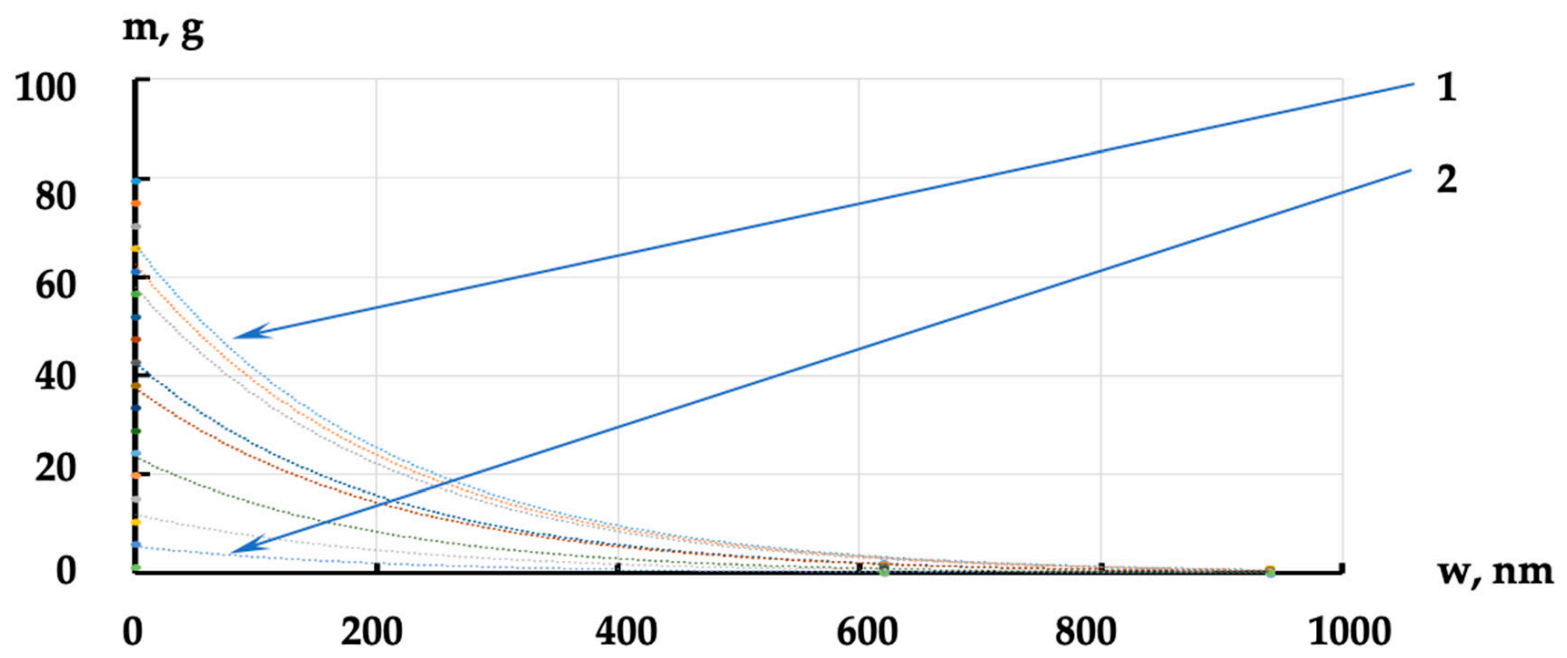
The model dependencies of n-heptane permeate mass m, g, on the thickness w, nm, of the PET-membrane transition layer for different n-heptane exposure times (from 5 (2) to 90 (1) min).

The total amount of data (Figure 11) is formally sufficient to build the simplest correlation-regression model since several samples with different SEM image divisions into the representative fragments were analyzed. For example, the entire set of exponential curves (Figure 13) (each of which describes the dependency of the permeability on the transition layer thickness at the concrete n-heptane exposure time) can already be quantified using least-squares-estimated averaged parameter values at a near-acceptable level of statistical confidence ($R^{2}≅0.8$).

Thus, we have demonstrated the rough dependence of the functioning efficiency on the morphology parameter for the fluorine- and oxygen-containing gas mixtures modified PET membranes’ prototypes using the example of n-heptane permeability studying.

## 4. Conclusions

The research made it possible to develop and test a technique for the quantitative analysis of polymer membranes’ physical structure based on the contour morphological heterogeneity functions calculations. The paper demonstrates the applicability and versatility of the proposed approach, taking into account the sufficiency of the considered scale ranges to describe the architecture of most existing polymer membranes and filters.

It was shown using the examples of PET-fiber-made nonwoven fabrics, LDPE packaging film, polysulfone, polytetrafluoroethylene and polyimide membranes that the contour morphological heterogeneity functions’ parameters are correlated with the key characteristics of the technology and the architecture of industrially manufactured separating items. This makes it possible to unify and algorithmize the procedure of allocating the functional zones in the structure of filtration equipment components (avoiding the use of measuring equipment operators’ subjective assessments). This ensures the repeatability and statistical reliability of the structure quantitative analysis results for the industrial samples and the experimental laboratory-made prototypes.

The integration and normalization allowed the construction of a specific form of contour morphological heterogeneity function in which it becomes probabilistic-like. It greatly simplifies the experimental samples’ SEM image analysis results’ interpretation. The developed algorithm for identifying membrane layers’ boundaries is based on determining the typical changes in the CMHF shapes. And it makes the metrological process reproducible and standardized.

The well-known spectral methods (e.g., based on Fourier transforms) effectively identify periodic features in the surface topography of the membranes and filters. The topological data analysis (TDA) based on persistence diagrams and homological descriptors allows for the classification of morphological inhomogeneities in terms of fractal geometry but requires additional specialized interpretation to address standard material science challenges. The proposed CMHF approach provides a simple tomographic characteristic that reflects the distribution of the total length of the sample structure inhomogeneities’ contours according to the average pixels’ brightness level (related to the distance from the effective surface to the characterized layer) of the corresponding SEM (or optical) image of the membrane or filter.

The CMHF approach standardizes the metrological procedure by relating the parameters of contour morphological heterogeneity functions to the architecture of multilayer membranes (unlike traditional image analysis techniques based on manual selection of measurement points and subjective identification of layer boundaries). In particular, the number of local extrema and inflection points of the CMHF and/or ICMHF can be used as the objective markers for determining the “surface”, “transition” and “bulk” components of the composite items. It reduces the level of subjectivity in interpreting the actual measurement results.

The practical significance of the proposed methodology is to eliminate the need for manual selection of measurement points and subjective interpretation of the results in production and scientific laboratories. The technique allows quantifying the thickness of the surface layer and the degree of its uniformity, taking into account the spatial distribution of the structural inhomogeneities. So it can be integrated into computer vision systems for automated quality control of membranes and filters directly on the production line.

The prospects for the research development include testing the applicability of the proposed approach to characterize the structure of membranes and filters made of other (including ceramic and metal) materials. It is also planned to supplement the analytical apparatus in the future by taking into account such complex parameters of the material structure as porosity and anisotropy. This will make it possible to create more accurate models of items’ architecture, which is necessary both to predict the properties and to ensure quality control.

## Figures and Tables

**Table 1:** The results of the 8-level Gaussian digital model n-threshold binatization.

n	1	2	3	4	5	6	7
$b_{k}\mathrm{dd}(x,y)$	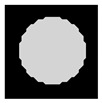	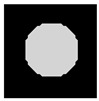	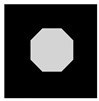	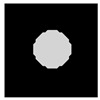	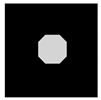	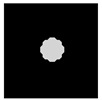	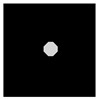
$l_{k}\left(n\right)$	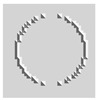	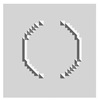	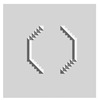	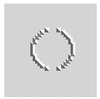	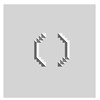	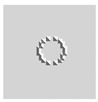	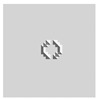
$\lambda \left(n\right)$	0.3625	0.2875	0.2375	0.2125	0.1625	0.1375	0.0875

**Table 2:** The results of the 8-level 3-Gaussian digital model n-threshold binatization.

n	1	2	3	4	5	6	7
$b_{k}(x,y)$	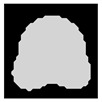	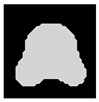	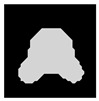	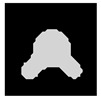	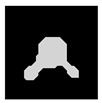	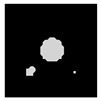	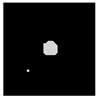
$l_{k}(n)$	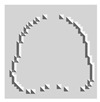	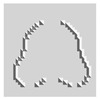	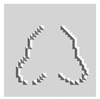	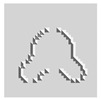	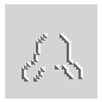	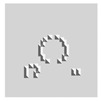	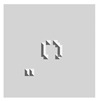
$\lambda \left(n\right)$	0.425	0.3750	0.3500	0.3375	0.3000	0.2250	0.1125

**Table 3:** The results of polysulfone-based hollow fiber membrane surface layer direct measurements.

№	1	2	3	4	5	6	7	8
Value, μm	69	78	65	70	70	78	85	85
**№**	**9**	**10**	**11**	**12**	**13**	**14**	**15**	**16**
Value, μm	85	90	85	79	78	82	104	68

**Table 4:** The results of transition layer between the sulfonated polytetrafluoroethylene and polyvinylene fluoride copolymer substrate direct measurements.

№	1	2	3	4	5	6	7	8
Value, μm	0.8	0.8	0.8	0.8	0.9	1.0	1.0	1.3
**№**	**9**	**10**	**11**	**12**	**13**	**14**	**15**	**16**
Value, μm	1.2	0.9	1.0	1.3	1.1	1.0	1.0	1.0

**Table 5:** The results of polyimide-based hollow fiber membrane surface layer direct measurements.

№	1	2	3	4	5	6	7	8
Value, μm	257	284	252	252	257	275	293	216
**№**	**9**	**10**	**11**	**12**	**13**	**14**	**15**	**16**
Value, μm	261	234	234	216	216	239	234	-

## Data Availability

Data are contained within the article.
